# Experiences of a transdiagnostic group, the Take Control Course, for clients with common mental health problems: A qualitative study

**DOI:** 10.1002/cpp.2303

**Published:** 2018-06-26

**Authors:** Lydia Morris, Warren Mansell, Rebekah Amos, Dawn Edge

**Affiliations:** ^1^ University of Manchester School of Psychological Sciences Manchester UK

**Keywords:** anxiety, depression, group therapy, mindfulness, primary care, transdiagnostic

## Abstract

**Objectives:**

Despite the promising effectiveness findings for transdiagnostic groups, studies have not explored clients' experiences. There is a risk that clients could perceive that the content of transdiagnostic groups is not sufficiently tailored to their specific problems. Our aims were to examine whether a brief transdiagnostic group, the Take Control Course (TCC), was acceptable to participants and to explore participants' perceptions of psychological change.

**Methods:**

Qualitative data were collected via 12 semistructured, in‐depth interviews. Data collection and thematic analysis were concurrent and iterative.

**Results:**

Three superordinate themes were identified: “Style and format,” “Control and flexibility,” and “Change.” The flexible group format was appreciated, as participants felt able to engage at their own pace and adapt relevant aspects. Greater clarity regarding what was within participants' control reduced distress and enabled effective pursuit of valued goals. Participants described significant (predominantly gradual) changes, including substantial improvements within relationships.

**Conclusions:**

The transdiagnostic format did not prevent participants experiencing the TCC as individually relevant. The flexibility and consistent theoretical framework seemed to contribute to this. The results indicated that greater consideration of control and mindfulness allowed greater cognitive flexibility, an ability to reprioritize and let go of unhelpful habits, which better enabled participants to meet their goals. Implications for group therapy include (a) clearly explaining the format of such groups to clients and (b) providing flexibility in the way the group is delivered where possible. Additional qualitative studies of transdiagnostic groups are required to establish if themes generalize to other transdiagnostic groups.

**Key Practitioner Message**

- This is the first detailed qualitative exploration of an explicitly transdiagnostic group.
- Findings indicated that a brief transdiagnostic group, the Take Control Course, was acceptable.
- Many participants found a greater understanding of control and awareness helpful in developing flexibility and pursuing important goals.
- The flexible delivery of the Take Control Course was valued and could be incorporated into other interventions.

## 1 INTRODUCTION

Common mental health problems, such as anxiety and depression, are often treated exclusively in primary care settings (Aillon et al., 2014). Due to high prevalence, cost‐effective psychological therapies are required that can meet such demand (Richards, 2010). One response has been offering psychological interventions in varied modalities, such as bibliotherapy, computerized, and group therapy (e.g., Knowles et al., 2014; Lewis, Pearce, & Bisson, 2012). An example of this is the Improving Access to Psychological Therapies (IAPT) programme in the United Kingdom. IAPT follows a stepped care model whereby the “least restrictive” treatment option is offered while still providing a treatment that is likely to lead to significant improvement (Bower & Gilbody, 2005). According to this model, clients with common mental health problems will often be offered brief interventions delivered using varied modalities, described as “low‐intensity interventions” (Bennett‐Levy, Richards, & Farrand, 2010). There have been concerns regarding whether low‐intensity interventions are acceptable to clients (Chambers, Haim, Mullican, & Stirratt, 2013; Sanders et al., 2012).

Transdiagnostic interventions have also been proposed to contribute to more effective service delivery, for example, by addressing multiple and co‐morbid problems (Andersson & Titov, 2014). They target cognitive and behavioural maintenance processes shown to maintain distress across disorders (Mansell, Harvey, Watkins, & Shafran, 2009). A meta‐analytic review of transdiagnostic interventions for anxiety and depressive disorders found large posttreatment reductions in both anxiety and depression symptoms, which were maintained at follow‐up (Newby, McKinnon, Kuyken, Gilbody, & Dalgleish, 2015). However, there has been limited qualitative research into transdiagnostic groups. There are a number of qualitative studies of mindfulness‐based interventions, which can be offered in a transdiagnostic format (Newby et al., 2015). But the majority of these qualitative studies focus on disorder‐specific groups or clients with severe physical health problems (Malpass, Mansell, Emsley, et al., 2012), and therefore, it is unclear if they generalize to transdiagnostic psychological health samples.

Qualitative studies of “low‐intensity” computerized therapy (Knowles et al., 2014) and self‐help interventions (Khan, Bower, & Rogers, 2007) indicate that client experiences can be negative. Findings include clients feeling that not enough time was spent discussing their specific problems and that interventions were not sufficiently tailored to them (Khan et al., 2007; Macdonald, Mead, Bower, Richards, & Lovell, 2007). Positive experiences were also described, such as feeling empowered (Khan et al., 2007; Knowles et al., 2014). A key example of low‐intensity groups is the cognitive–behavioural therapy (CBT)‐based Stress Control course for Generalized Anxiety Disorder (White, Keenan, & Brooks, 1992), as recently, it has been suggested that this course is transdiagnostic because it works on negative affect (Barlow, Allen, & Choate, 2004; White, 2010). Average satisfaction ratings are high for both Stress Control (Burns, Kellett, & Donohoe, 2016) and Take Control Course (TCC; Morris, Mansell, Emsley, et al., 2016), but detailed qualitative understandings of participants' positive (and negative) experiences are lacking. Therefore, examining acceptability remains important in establishing whether low‐intensity and transdiagnostic groups meet the needs of the target population (Ayala & Elder, 2011).

The current qualitative study reports interview data from participants who accessed a brief (six‐session, with average session length of 1 hr) transdiagnostic group: the TCC. The study aims were to examine (a) participants' experience of the course, such as their experience of the modality and the length of the intervention, and (b) participants' perceptions of psychological change and which elements of TCC contributed to this. The first research aim reflects the concerns already detailed regarding the acceptability of low‐intensity interventions. The second research aim responds to the current lack of qualitative data regarding psychological change processes within transdiagnostic groups. We now turn to the scientific background for this aim.

Despite its transdiagnostic group format, the TCC is adaptive to the particular needs of individual clients. Weekly feedback is collected to facilitate adaptation. The core content of each session is prescribed, but certain techniques reoccur so that subsequent implementation of techniques can be adapted in accordance with feedback. Further, within the majority of sessions, there are optional components that can be delivered if these are of interest to clients and time allows; this also allows flexibility where discussion is proving valuable. Given that the TCC is not diagnosis‐specific, it is important to establish whether clients perceive that content is tailored to them.

The TCC is derived from the principles of perceptual control theory (PCT; Powers, 1973). PCT proposes that control is central to well‐being (Powers, 1973). To implement control, current perception is constantly compared with internal references, and actions occur to minimize discrepancies from these references (Powers, 1973). When important perceptions are not sufficiently close to their desired internal reference, then this is described as “loss of control” (Morris, Mansell, & McEvoy, 2016) and can entail psychopathology if control is not restored (Alsawy, Mansell, Carey, McEvoy, & Tai, 2014). Therefore, the TCC focuses on enabling participants to understand the process of control.

PCT also specifies that internal references are arranged hierarchically with the higher levels, setting the reference values for lower levels (Powers, 1973). For example, the higher level reference “be a good friend” sets the reference “keep in regular contact,” which could lead to actions such as texting friends and meeting regularly. Higher level references often represent longer term goals and values. Enduring conflict between higher level references particularly entails loss of control and psychopathology (Alsawy et al., 2014).

Awareness of such higher level conflict is required to regain control. PCT specifies a mechanism by which changes to an individual's control system occur. This mechanism is a trial and error process termed *reorganization* (Powers, 1973). Changes occur at the point that awareness is directed within the control system. It is therefore of vital importance that awareness is directed towards the appropriate level of the hierarchy. PCT‐based accounts of significant change generally predict reports of sudden changes, or “insight moments,” because the reorganization process will at some point generate effective change, which is often experienced as a shift in perspective (Gianakis & Carey, 2011). However, it is possible that change is experienced in a more gradual way because the physical manifestation of higher level changes will often be expressed through lower level systems. For example, Chantelle had a conflict between “I need to express how I feel in relationships” and “I must not upset other people.” She was often finding it difficult to meet both her goals, and there were a couple of relationships she was feeling particularly frustrated within. She began to see a therapist and started trying out different ways of managing her frustration; she started expressing herself more (a lower level attempt to meet one of her goals) but felt guilty and uncomfortable. Following a “breakthrough” in the therapy, she expressed herself without experiencing this guilt and discomfort. There was an important change in her perspective, but because she had already been making changes, it did not seem sudden or dramatic.

Given that enduring higher level conflict is specified as the primary contributor to symptoms of psychological distress, the TCC focuses on enabling “awareness of valued higher‐level goals (and reasons for change); and awareness of higher‐level goal conflict” (Morris, Mansell, & McEvoy, 2016, p. 8). Accordingly, a key reason for using awareness/mindfulness techniques within TCC is enabling clients to develop present moment awareness that can be brought to bear in resolving conflicting goals. Mindfulness techniques are also important within TCC as evidence indicates that they can promote cognitive flexibility (Chiesa, Calati, & Serretti, 2011; Gu, Strauss, Bond, & Cavanagh, 2015; Shapero et al., 2018). Within PCT, *flexibility* as a cognitive process refers to flexibility of awareness. For example, “flexibility of awareness” would be indicated both by an individual having sufficient cognitive flexibility to be aware of relevant higher level goals *and* being able to implement these via lower level goals. The two key elements here are a broad awareness of higher level goals (including goals that could potentially conflict) in conjunction with a mobility of awareness, so that an individual does not “get stuck” at higher level abstract goals but is able to implement higher level reorganization using lower level goals (Morris, Mansell, & McEvoy, 2016). Behaviourally, this might be demonstrated by the ability to disengage from patterns of responding that are no longer helpful in meeting an individual's important goals (Morris & Mansell, in press). Varied strands of evidence indicate that mindfulness can enable a broad and mobile awareness that allows access to conflicted goals and distressing experiences. For example, mindfulness techniques can enable observation and tolerance of internal experiences that are uncomfortable or distressing and indicative of conflict (Farb, Anderson, Irving, & Segal, 2014; Lippelt, Hommel, & Colzato, 2014). This allows breadth of awareness, as distressing material is not excluded. Through sustained awareness of such experiences, the individual's perception is likely to become more elaborate and deep‐rooted conflicts more likely to be resolved (Alsawy et al., 2014; Teper, Segal, & Inzlicht, 2013). Further, Teper et al. (2013) have proposed that mindfulness enhances conflict monitoring and allows individuals to remain more open to conflicts: “Thus, mindfulness promotes executive control by enhancing experience of and attention to transient affects—the control alarms—that arise from competing goal tendencies” (Teper et al., 2013, p. 452).

TCC specifically targets PCT change mechanisms, and therefore, it is expected that this would be reflected in participants' accounts. However, PCT specifies mechanisms of psychopathology and change that are common across disorders, and so other therapies are likely to also address these processes to varying degrees (Morris, Mansell, & McEvoy, 2016).

## 2 METHODS

### 2.1 Study context

This exploratory, qualitative study was embedded within a 12‐month randomized parallel group trial to establish whether the TCC was noninferior to individual low‐intensity CBT (*N* = 156). The study was approved by the North West‐Greater Manchester East Research Ethics Committee.

### 2.2 Setting

All participants were recruited from Salford (North West of England). Ranked among the 10% most deprived local authority areas in England (Department of Communities and Local Government, 2011), 92% of Salford's population are of White ethnicity (Office for National Statistics, 2011).

### 2.3 Participants

Recruitment to the trial was from Salford Six Degrees Social Enterprise (formally the Primary Care Mental Health Team, Six Degrees are the main provider of low‐intensity IAPT services in Salford). Adults aged 16 and above who were suitable for low‐intensity services were recruited. This included individuals with mild–moderate depression and anxiety that emerged within the 12 months prior to referral. However, other mild–moderate problems are addressed within this service context, and clinical decision making is according to stepped care models. Other factors that were considered included level of suicidality and the extent to which problems impaired functioning. Sufficient English language skills to understand TCC materials were also required (i.e., the verbal and written language abilities required to read and complete simple worksheets and understand verbal presentations).

Participants' demographic and psychiatric characteristics are reported in Table 1 and are comparable with the overall randomized controlled trial sample (e.g., average age is the same and both male and female participants are represented in the current sample). Table 2 provides similar characteristics for individual participants. Pseudonyms are used to maintain confidentiality. Specific ages of participants are not detailed to further protect confidentiality and because age had no noticeable influence on the results. Participants' pretreatment anxiety and depression scores ranged from mild to severe (see Table 2 for severity levels). Participants engaged in one of eight different TCCs and had a range of different levels of improvement at time of interview. Although the entire sample was White, this is representative of the 95% of White participants who accessed the TCC. The average number of sessions attended was higher in the current sample (*M* = 4.8) than the randomized controlled trial sample (*M* = 2.2). Participants who accessed one to two sessions were given every opportunity to participate, but all declined. Reasons for nonparticipation varied, but being “too busy” was most frequently cited.

**Table 1:** Participants' demographic and psychiatric characteristics

Demographic and psychiatric characteristics		
Female, no. female (%)	7 (58.3)	
Ethnicity, no. White British or Irish (%)	12 (100)	
Age, *M* (*SD*)	41.5 (14.1)	
Average number of sessions attended, *M* (*SD*)	4.8 (1.1)	
	Baseline (prior to TCC)	6‐month follow‐up
PHQ9 scores, *M* (*SD*)	13 (6.6)	9.7 (9.1)
GAD7 scores, *M* (*SD*)	12 (5.4)	8.1 (6.7)

*Note*. PHQ9: Patient Health Questionnaire Depression Scale; GAD7: Generalized Anxiety Disorder Questionnaire; TCC: Take Control Course.

**Table 2:** Individual participants' demographic characteristics and presenting problems

Name (pseudonym)	Gender	Number of sessions	PHQ9 scores^a^		GAD7 scores^a^		Presenting problem (identified by clients prior to therapy)	Individual therapy
			Pre	6‐months	Pre	6‐months		
Sarah	F	3	22 (Severe)	27 (Severe)	19 (Severe)	18 (Severe)	Problems with Mum's health issue and myself feeling depressed and unwell.	Yes (previous)
James	M	5	4 (Subclinical)	3 (Subclinical)	7 (Mild)	3 (Subclinical)	Wanting to go places (holiday) but unable due to anxiety. Expressing feelings to people.	Yes (previous)
Robert	M	3	8 (Mild)	5 (Mild)	12 (Moderate)	10 (Moderate)	Anxiety/physiological problems deriving from stress. Inability to switch off mentally/concern regarding how others view me.	Yes (previous)
Laura	F	6	20 (Severe)	17 (Moderate–severe)	20 (Severe)	21 (Severe)	Too many tasks, too little time. Lack of support, I help everyone else; they are reluctant to help me.	No previous therapy disclosed
John	M	6	12 (Moderate)	10 (Moderate)	8 (Mild)	4 (Subclinical)	Workplace colleague. Having enough work for shop floor.	No
Claire	F	5	10 (Moderate)	6 (Mild)	11 (Moderate)	4 (Subclinical)	Menopause. Work: upcoming department restructure.	No
Rachel	F	4	13 (Moderate)	2 (Subclinical)	15 (Severe)	4 (Subclinical)	Anxiety due to problems with social workers and social services (children taken away). Multiple bereavements.	Yes (previous)
Mike	M	6	25 (Severe)	25 (Severe)	16 (Severe)	15 (Severe)	Depression. Anxiety.	Yes (previous)
Anna	F	5	11 (Moderate)	2 (Subclinical)	4 (Subclinical)	2 (Subclinical)	Unemployed. Feel like a failure due to not being employed.	No
Catherine	F	6	6 (Mild)	14 (Moderate)	5 (Mild)	11 (Moderate)	Relationships with family and friends. Relationship with partner.	Yes (previous)
Christina	F	5	8 (Mild)	2 (Subclinical)	10 (Moderate)	2 (Subclinical)	Difficulty making friends and family relationships. Anxiety.	Yes (previous)
Dave	M	4	17 (Moderate–severe)	3 (Subclinical)	17 (Severe)	3 (Subclinical)	Constant worry and overthinking. Feeling as though there is something wrong with me.	Yes (subsequent)

*Note*. PHQ9: Patient Health Questionnaire Depression Scale; GAD7: Generalized Anxiety Disorder Questionnaire.

a The PHQ9 and GAD7 can be used to determine the likely severity levels of depression and anxiety (Richards & Suckling, 2009). For the PHQ9, these are as follows: not depressed 0–4; mild 5–9; moderate 10–14; moderate–severe 15–19; severe 20–27. For the GAD7, these are as follows: 0–4; mild 5–9; moderate 10–14; severe 15–21 (Richards & Suckling, 2009).

### 2.4 Interview schedule and data collection

Interviews were conducted within general practitioner (GP) practices and health centres in Salford. Data were collected by a Masters student and Doctoral student (first author) using a semistructured interview schedule. The interview schedule was developed based on previous qualitative research with similar aims (e.g., Finucane & Mercer, 2006) and through discussions within the research team. The interview schedule was piloted opportunistically; volunteers who had received a therapeutic intervention in the past gave feedback on the wording and relevance of questions. Based on feedback from pilot interviews and liaison with the research team, the interview schedule was amended and a final version was developed. Changes made in response to pilot interviews included changing the structure of the interview schedule to make it easier for researchers to cover all relevant themes and changing question wording and adding additional prompts (especially where questions could have been answered yes/no, or did not result in relevant answers from volunteers). Interviews ranging between 30 and 60 min were conducted and were digitally recorded. In addition to the questions on the interview schedule, interviewers asked probe questions, which were integral to the interview schedule. These were designed to encourage participants to elaborate on comments and to obtain more details regarding their experience of TCC and perceptions of its impact. The interview schedule is included in Appendix A.

Interviews were conducted shortly after 6‐month follow‐up wherever possible (average time since follow‐up was 39 days; included in this is one client that was uncontactable at 6‐month follow‐up, and for this client, the measures were taken 9 months after baseline appointment). Participants were interviewed at this time to provide an indication of whether techniques and understandings were used to maintain change after the sessions had finished.

Interviews were conducted until saturation of themes occurred (Guest, Bunce, & Johnson, 2006). Saturation is defined in terms of “the point in data collection and analysis when new information produces little or no change to the codebook” (Guest et al., 2006, p. 65). Data collection and analysis were concurrent and iterative thereby enabling evaluation of whether saturation has been reached (Tuckett, 2004).

### 2.5 Analytical strategy and procedure

Analysis followed Braun and Clarke's (2006) six‐phase approach to thematic analysis within a critical realist framework (Creswell, 2009). See Table 3 for details.

**Table 3:** Braun and Clarke's (2006) six‐phase approach to thematic analysis, including detail of how this was implemented and by whom

Phase	Application of the phases within this study
1. Becoming familiar with the data	The first author conducted the majority of interviews and transcribed the data. Transcripts were repeatedly read, and particular attention was paid to rereading transcripts of interviews that the first author had not conducted.
2. Generating initial codes	The first author coded the data in a systematic fashion across the entire dataset. All interview data that related to the TCC were coded.
3. Searching for themes	Data were coded using NVivo 10 (QSR International's NVivo 10 Software, 2014) to support data management. All significant patterns in the data were noted and initial table of second‐order codes and quotes created. Throughout this and subsequent stages, findings were reviewed for coherence and credibility by D. E. and W. M. and the raw data regularly referred to.
4. Reviewing themes	From the initial table of significant second‐order codes and discussions with D. E. and W. M., candidate themes were identified. These were then refined by referring back to data and codes and by creating a detailed thematic map. Candidate themes were examined to establish whether they were coherent, externally heterogeneous, and had explanatory power.
5. Defining and naming themes	Through examination of the detailed thematic map and further discussions, a more parsimonious list of themes were created. These were refined through peer debriefing and verification with Dr Noke and through a member‐checking group. Dr Noke is a qualitative researcher who was not involved in the study team or PCT research.
6. Producing the report	The report was drafted and feedback obtained from D. E. and W. M.

*Note*. TCC: Take Control Course; PCT: perceptual control theory.

Thematic analysis was conducted by the first author and is described in some detail to provide evidence of the trustworthiness of the study, specifically the credibility (internal validity) and dependability (reliability) of findings (Shenton, 2004). Furthermore, exact numbers of participants who reported content relevant to a particular theme are quoted throughout the results section. Although the appropriateness of such numerical specificity is debated within the qualitative literature, the authors believe that in the context of the currentstudy it provides a helpful indication of how representative a theme is by detailing the number of participants that expressed a certain experience or perspective (Braun & Clarke, 2006). Methods of establishing reliability and validity used within quantitative research are often not applicable to qualitative data (Creswell, 2009). However, measures can be taken to establish the credibility and dependability of qualitative data (Shenton, 2004). Peer verification, regular debriefing, and member checking were used to enhance trustworthiness and confirmability. Member checking confirmed that the themes identified were congruent with participants' experiences (Carlson, 2010). A key informant's approach (Onwuegbuzie & Leech, 2007) was used so that feedback could be obtained from participants who gave both positive and critical feedbacks and who had a range of improvements in presenting problems. Although there were a range of participant experience represented within the feedback session, there were slightly more people who felt they had benefitted from the course. This was representative of the sample as a whole. However, attempts were made to invite additional participants who provided critical feedback or described not being sure that the course was beneficial to them.

Criticisms have been made of member checking, such as that themes are abstracted and therefore may not be easily recognizable as participants experience and that participants may have changed their mind since they were interviewed (Angen, 2000; Morse, Barrett, Mayan, Olson, & Spiers, 2002). Further, for nonrealist frameworks, it is argued that qualitative accounts cannot capture a truth that can then be verified with participants (Angen, 2000). However, it is argued that what is being established during member checking is whether participants believe that the themes sufficiently represent their experience. It is suggested that whether participants feel the research represents their experience is of considerable importance because (a) their words and general experience are what is being captured within the research; (b) the account is created within an ongoing “dialogue” between participant interviews and researcher interpretations and if participants have changed their minds this is useful information to capture; and (c) in the case of intervention studies, such as this one, the intervention needs to be of relevance to potential clients and so it is helpful if experiential accounts are also of relevance to actual clients (Cho & Trent, 2006).

Reflexivity in relation to the researchers' background experiences and perspectives is useful in order to consider the potential influence of these on interpretation of the data (Carlson, 2010). The first author has greater experience of working within some conceptual frameworks, such as cognitive–behavioural theories and PCT, and is a Clinical Psychologist. The first and second authors are both involved in research into PCT and developing interventions based on PCT. Both authors therefore have a particular interest in this theory. The first author is a practicing Buddhist and has specifically trained in mindfulness‐based interventions.

### 2.6 Intervention

The TCC is six sessions, and the key topics covered in each session are outlined here (see Morris et al., in press, for a detailed description of the TCC and how to deliver it). *Session 1* introduces the central theme of control. The overall objective is to enable clients to understand what is meant by “control” and to encourage them to consider the things that they can, and want to, have more control over. *Session 2* examines processes and ways of responding that can block control, focusing particularly on thinking styles such as rumination, worry, and self‐criticism. A key message here is that individual thoughts, even “negative” ones, are not the problem. Worry, self‐criticism, and rumination are normal processes that most people engage in. They become problematic only to the extent that they impede important life goals or limit a person's awareness of these goals. The principle of a hierarchy provides a key basis for *Session 3*. The fundamental message of Session 3 is that sometimes individuals' attempts to control their emotions, avoid distress, or keep safe can actually prevent them doing things they consider really important. This encourages clients to focus on their longer term (higher level) goals and to consider how to prioritize these over short‐term goals that are getting in the way. *Session 4* encourages clients to take control of environmental factors. This session emphasizes that there are many things in the environment that can be changed to make the world more like they want it to be, while acknowledging that there are also some things over which they will have limited control. Session 4 focuses on helping people take a wider perspective on their own needs and balancing them with those of others who are important to them. The focus of *Session 5* is to encourage participants to recognize the strengths, qualities, and resources they have, especially at times when things are difficult for them. *Session 6* focuses on consolidating what has been learnt and planning for the future. Clients choose elements of the TCC that they want to revisit during this session.

A number of techniques and understandings are introduced within the TCC, two of which are detailed here to exemplify how the processes described in the introduction are targeted. One of these is a goal‐focused exposure exercise that involves imaginal exposure to an uncomfortable experience (e.g., social situation and feeling of failure), but not one that will be extremely uncomfortable (Carryer & Greenberg, 2010). In initial sessions, participants are strongly encouraged to choose the mildest experience. The way in which this exercise is framed is in the context of meeting important goals in order to promote awareness of goal conflict. Another technique, which is usually delivered prior to the goal‐focused exposure, is brief awareness (including mindfulness meditation). Use of such techniques can also support clients to sustain attention on problematic experiences indicative of goal conflict (e.g., during imaginal goal‐focused exposure; Morris, Mansell, & McEvoy, 2016).

## 3 RESULTS

Three superordinate themes, “Style and format,” “Control and flexibility,” and “Change,” and 10 subordinate themes were identified (see Table 4). These are described in detail below. Participant quotes are used to exemplify the themes. Table 5 details the themes and subthemes and the number of participants who referred to these.

**Table 4:** Superordinate and subordinate themes

Superordinate theme	Subordinate theme
Style and format	Flexibility and “at ease” environment Supporting each other Better than one‐to‐one “The right length of time”
Control and flexibility	There are things I can and can't control Pursuing what really matters Mindfully interrupting unhelpful responses
Change	Pace of change Practice, integration, and consolidation Improved relationships

**Table 5:** Themes and subthemes and the number of participants who referred to these

Theme	Number mentioned by	Subordinate theme	Number mentioned by	
			(exc negative cases)^a^	Negative cases^b^
Style and format	12	Flexibility and “at ease” environment Supporting each other Better than one‐to‐one “The right length of time”	7 8 6 8	2 2 1
Control and flexibility	11	There are things I can and can't control Pursuing what really matters Mindfully interrupting unhelpful responses	8 9 6	2 (this understanding not particularly helpful) 3 (difficulties with mindfulness exercises)
Change	10	Pace of change Practice, integration and consolidation Improved relationships	7 10 6	

a This column refers to the number of participants that expressed at least one comment or utterance in line with the overall subordinate theme. For example, they expressed appreciating the flexible format of the group, or that they thought the length of the sessions was good.

b The negative cases column refers to when participants commented on the overall subordinate theme but expressed a very different sentiment from the majority of participants. For example, they would have preferred a less flexible format, or they would have preferred a longer session length.

### 3.1 Style and format

There were a number of aspects of the style and format of the TCC that participants identified as helpful. These helpful elements were organized as subthemes, and these are regarding flexibility and the “at ease” environment, appreciating the group format, supporting each other, and generally appreciating the brief format.

#### 3.1.1 Flexibility and an “at ease” environment

The TCC and the facilitators offer a range of strategies and encourage participants to “take what is helpful for them.” In addition, the content of the group is adapted around the participants' needs. Seven participants valued this. Christina described the flexible style as providing permission for her to become more flexible and to adapt what they learnt to her life.

I've learnt to integrate the things that we've learnt into my life more. Umm, not necessarily exactly how they done on the course but I did feel like that was OK …. Because sometimes you feel, “this is the way to do it,” “don't do it that way you're doing it wrong,” where as it didn't feel like, it felt like take a little bit of anything that you want and, if it works for you, great!

Although not all participants made the explicit link between the course modelling flexibility and them being flexible, seven described adapting “techniques” that enabled them to apply these more directly to everyday life.

There was a lady there who said that the train thing helped her. Well that didn't work for me, thinking let them *[thoughts]* pass me by. I had to tell myself “no, stop thinking about that now. That was then, this is now.” (Catherine)

(Note: The train metaphor is used to exemplify ways of responding to thoughts [and other experiences]. The suggestion is that participants can let thoughts pass them by, like train carriages passing through a station. Catherine describes a more proactive attitude to her thoughts; she consciously tells herself to stop thinking these thoughts rather than just letting them pass.)

Five participants described appreciating the informality and “at ease” (Anna) environment of the course. The flexibility seemed to contribute to this as they described finding the focus less intense as “there's no pressure” as “in a group the spotlights moves around” (Rachel).

It was very informal, it wasn't kind of like some AA meeting where you stand up and you're forced to talk and give your story or anything. (Anna)

However, there were two participants (John and Laura) who had reservations about the flexible style, and their comments suggested that they would have preferred something more directive and more of a didactic style. Both accessed the same TCC.

John: There was a lovely lady there, but it just seemed very softly presented.

Interviewer: Hmm, when you say “softly presented,” like it didn't quite hit home, or?

John: Like, I think it just needed a little bit more telling to the people.

I think I got more out of that style, like teaching, cus it was quite a bit like a teaching course I think. (Laura)

Both participants recognized that the flexible style might have been due to the necessity of accommodating different people:

That's the thing though, you've got be so careful for everybody's individually in a different place …. So you can understand, you've got to aim it at everybody and probably by opening it on the softer one, that's what they're trying to do. (John)

#### 3.1.2 Better than one‐to‐one

All participants had accessed at least two individual assessment sessions. Eight of the 12 had accessed individual therapy prior to the qualitative interview. Six participants expressed they preferred the group format and felt that they had made more progress in the group than they would have done in one‐to‐one.

Yeah, I don't think that would have benefited *[from one‐to‐one]*. It was really nice having other people chip in, not feeling like you had to speak …. Almost have that time to sit there and assess how you feel about it, and for it to work on you, instead of it being forced out of you, which one‐to‐one by definition does. (Anna)

Where they were able to identify a reason for this, they attributed it to aspects of the style of the TCC described in previous themes, for example, that the group felt less formal than individual therapy, and they were able to pace their disclosure and learn from others.

I think for me, cause of the way of me, being shy and everything. Being in the group it's more like other people can do the talking and I can take in stuff about what they might have gone through, and how things are helping them as well. (Mike)

Although there was strong support for a group approach, there were two participants who preferred individual therapy; this was generally attributed not only to the format but also to the nature of the problems that they wanted to work on.

I think it was just because I felt it *[one‐to‐one]* was a bit more personal. (Dave)

And I do think, maybe there is great value for me in a one‐to‐one environment rather than the group session, not to undermine anyone else's issues, but because of that specific thing that I could see that their issues—I think—were rooted to a specific event that occurred recently. (Robert)

#### 3.1.3 Supporting each other

Eight participants identified that they had benefitted from being with others. Feeling able to share without being judged and hearing the perspectives of others contributed to the “normalization” of their experience.

So I thought, “I'm not going crazy here, that's good.” There are other people who—for various reasons—are going through the same things. (Anna)

However, participants also identified that it was really important that they did not feel “pushed” (Catherine) to share and could do so at their own pace.

It was helpful cause it wasn't, it wasn't, intrusive …. You didn't have to elaborate on any of your issues really it was just the techniques. Even then you could just say that they were helping … you didn't need to elaborate. (Catherine)

The fact that that the TCC does not rely on self‐disclosure supported participants to feel comfortable in the group. Two participants expressed specific concerns that the TCC would rely heavily on self‐disclosure, such as an Alcoholics Anonymous group. Two others expressed fears around opening up to others. They expressed relief that self‐disclosure was not required.

I didn't have much hope for it really, cause you have this stigma that you're all going to be sat in a circle relaying everything, telling all your business. So I was delighted it wasn't like that. (Catherine)

#### 3.1.4 “The right length of time” (Catherine)

Eight participants expressed appreciating the hour‐long session length. For example, Dave said it was “an ideal length of time” and Sarah, who described difficulties with concentration, said “it wasn't too long because it was interesting.” Not all participants gave reasons for appreciating this length, but those who did said that they felt an hour enabled them to “work your day around it” (Anna) and was particularly convenient. Further, it was felt that it would be difficult to concentrate for longer.

The one person who would have liked longer sessions felt that they would have preferred about an extra half‐hour.

Just to maybe give the tutors the time to explain what each thing's about and how it's meant to work. (John)

Three participants expressed six sessions were enough, but there were three people who would have preferred more sessions (Laura, Anna, and Christina). The reasons for this varied including experiencing significant life events during the course, and so feeling more support was required (Laura) and wanting to “hammer home” (Anna) what had been learnt. Only one out of the three (Laura) described a strong need for more sessions, whereas the other two described a desire for consolidation existing gains:

I really enjoyed the experience of the course and the routine of it and I found it a very grounding experience in each week …. So I think it was as much that as anything else that. (Christina)

### 3.2 Control and flexibility

An increased understanding of control was a notable feature of nearly all participants' accounts and enabled clarification of the things in their lives that they could change to meet their goals. This flexibility is a form of cognitive adaptively and enabled participants to better meet their goals. Mindfulness was a key tool in enabling clients to pursue important goals and manage unhelpful responses more effectively.

#### 3.2.1 There are things I can and can't control

This understanding was described as helpful by eight participants and seemed to enable participants to target the things they could control in order to meet their goals.

So it made me realise things that I could have an impact on and things that I couldn't. (Christina)

It also seemed to support the majority to “let go” of dwelling on concerns that they could not control.

Yeah, and I think, going back to when I was thinking about worrying things, I'd think why do I need to worry about that, what can I do about that. Some things you can't do anything about. (Claire)

However, there were two participants who described not being able to “let go” of concerns that they were unable to control:

I couldn't say right I'm gonna take control of this, I'm not gonna worry …. I couldn't because when I came out of there my problems were still there. (Sarah)

These two participants described experiencing major life events or significant ongoing problems during the TCC, for example, a chronic physical health problem or a marked lack of social support. One participant described this as a helpful understanding overall because she could change her responses to stressors, but she also recognized she would not regain sufficient control without some change the work situation she was in

But erm, it still didn't take me out of the position that I was actually in, you know if you can't control people but you still have to work for them and with them, and you, you can try doing things differently and it doesn't actually go, to them anyway, and I was just stuck in one of those really low points. (Laura)

#### 3.2.2 Pursuing what really matters

Nine participants described increased clarity regarding what was really important to them and an increased flexibility regarding how they could pursue this. This included understanding what areas of their life they could influence (control) to achieve important goals.

“I mean you said then, was it session 3, about (I: long‐term goals) umm well, I'd keep doing negative things and I was expecting a different outcome and that's a waste of time …. And I realised I needed to stop.” (Catherine)

This ability to pursue what mattered to them was reflected in a significant perspective change in some. This could entail both identifying whether a concern or preoccupation was really important and identifying what could be done to meet important goals.

“Like I say, that upward arrow thing, that was just brilliant and the day she went like that, ‘it doesn't matter.’ I just thought, ‘why am I writing this?’ It's the same thing every week and you're right on the grand scale of things this doesn't matter.” (John)

(Note: The “upward arrow” technique physically resembles the downward arrow technique used within CBT (apart from the arrows are progressing upwards instead of downwards). However, the questions fundamentally differ, and therefore, the end point is clarifying the goals that are most important to the client. The questions used are as follows: Why is “X reason/goal” important to me, or why does it help?)

“Umm I think mainly it was being able to break everything down and not having to focus on controlling the bigger picture when you can focus on the smaller parts first, then build it up.” (Rachel)

#### 3.2.3 Mindfully interrupting unhelpful responses

Six participants described using mindfulness to increase their awareness in their day‐to‐day lives so that they could identify when they were getting into responses that did not help them meet important goals and interrupt these.

So I could then start to identify when I was getting into this negative thinking so I'd have to … “reign myself in really.” (Catherine)

As well as giving participants a “tool” to enable them to interrupt unhelpful responses, these six described using mindfulness to work more effectively with difficult experiences. Interestingly, very few participants described still using formal awareness (meditation) practices. This is likely to reflect the emphasis within the TCC on using mindfulness to process and respond to difficult experiences.

I mean today a song played at work, and 6 months ago—before the course—I would have had to leave the building, turn it over, be very distraught. It's got easier and easier, because I don't get stuck in the negative thinking …. Yeah, I try and then do some mindfulness techniques …. I don't do any mediation at all. (Catherine)

However, three participants described difficulties using mindfulness (Robert, Anna, and Sarah). These difficulties were particularly described in terms of problems disengaging from unhelpful or distracting thinking, but there were more general difficulties raised:

Difficult to put into practice on two levels, one because you are dealing with the embedded ways of behaving that are so engrained in you and two, you don't necessarily have the capacity to be able to get any time to step back. (Robert)

### 3.3 Change

Ten participants were still using understandings and techniques from the TCC 4–8 months after attending, and it seemed that these offered the potential to equip them to continue to maintain their wellbeing in the future.

#### 3.3.1 Pace of change

Certain theoretical accounts indicate that significant change is more likely to be experienced as either sudden or gradual change. Five participants described experiencing gradual, as opposed to sudden, change.

I feel like a lot of things happened on the course and then I felt that I was quite different at the end, but … it was gradual learning rather than here's all the information and just take it away …. Yeah it didn't feel that sudden but still quite profound. (Christina)

However, three participants described experiences of “light bulb moments” (Laura) or “clicking a switch” (Rachel) that suggest sudden moments of insight.

#### 3.3.2 Practice, integration, and consolidation

Ten participants described continuing to use understandings and techniques from the course. This was made possible by many of them consciously consolidating the material during the course and could contribute to a gradual cumulative experience of change.

I do the school run and had the paperwork from the sessions in the car. So I'd go to school, do the school run early and just be sitting reading everything. (Catherine)

As well as referring to the materials used within sessions, eight participants described revisiting techniques. They were then able to continue to put understandings into practice once the course had finished.

Interviewer: Is that *[supportive image]* something you've continued doing after you finished the course?

Mike: Quite a few times, I've sat and done something along that line

(Note: The supportive image is an imagery‐based technique that encourages participants to “create” and imagine an image that represents qualities that they value and believe will support them.)

Two participants also described a less conscious or effortful integration process.

And I probably wouldn't have remembered it happening but I do that an awful lot more now, think “really, is this anything to do with me, is there anything I can do?” …. I feel like I'm doing that process with a lot of things …. So that's obviously very well‐integrated cause I wouldn't have been able to tell you that, that I was doing that. (Christina)

#### 3.3.3 Improved relationships

Six participants described changes in how they related to other people. These changes were varied and significant. These included “expressing feelings more” (James), getting less irritable with others, engaging in less negative comparisons between themselves and others, and strengthening social networks in other ways.

I don't know cause, it's like I got into a new relationship as well …. But umm, whatever clicked, I didn't go for ones I'd normally go for. Cause I'd normally go for the big bad boys, total complete jerks, umm but no *X [name]* is completely CRB checked, works full time, everything else, completely supportive. (Rachel)

Based on the previous themes and the benefits that participants identified of being in a group context, it seems possible that the group built confidence in relating to other people; it is also possible that participants had the opportunity to clarify their interpersonal goals (e.g., during Session 4). However, this was not explicitly explored with participants during the interview.

## 4 DISCUSSION

Study aims were to examine (a) participants' experience of the course, such as their experience of the modality and the length, and (b) participants' perceptions of psychological change and which elements of the course contributed to this. The results indicated that TCC was generally acceptable to participants, as satisfaction and understanding levels were generally high. The “Style and format” themes indicated that participants valued the flexibility of the TCC and generally liked the brief format. Themes regarding “Control and flexibility” suggested that the theoretical threads of control and awareness were understood and utilized by participants. Further, the results indicated that this understanding allowed greater cognitive flexibility, in the form of an ability to reprioritize and let go of unhelpful goals and habits, which better enabled participants to meet their goals. “Negative cases” were present in most of the themes, which entails that there were one or two participants per theme who expressed a different experience from the majority. Therefore, a minority of participants would have preferred a longer or individual format, did not find a greater understanding of control particularly helpful, or did not find awareness techniques useful.

### 4.1 Participants' experience of the course

This is the first qualitative evaluation of the acceptability of a brief transdiagnostic group, targeted at clients with common mental health problems in a “low‐intensity” primary care setting. Qualitative explorations of both transdiagnostic and low‐intensity groups are limited. Therefore, information is lacking as to whether clients experience transdiagnostic groups as being sufficiently tailored to meet their needs or whether they express a preference for longer group interventions. Due to the lack of detailed qualitative data regarding transdiagnostic groups specifically, it is difficult to provide an informative comparison. However, there are two studies that provide some qualitative data (e.g., case studies and quotes from participants), which focus on group Unified Protocol (Bullis et al., 2015) and transdiagnostic group CBT for anxiety disorders (Norton & Hope, 2008). Both of these transdiagnostic group interventions demonstrate differences from the TCC, such as using techniques that challenge distorted thinking. Both are substantially longer than the TCC (i.e., twelve 2‐hr sessions). The Unified Protocol has a greater emphasis on the adaptive function of emotions than the transdiagnostic group CBT and is designed to target anxiety and mood disorders (Norton & Hope, 2008; Wilamowska et al., 2010). The qualitative and case study data from these studies are discussed below.

Earlier reviews of qualitative studies of low‐intensity interventions (computerized and guided self‐help) found common themes that not enough time was spent discussing participants' specific problems and that interventions were not sufficiently individualized (Khan et al., 2007; Knowles et al., 2014). This was not a common aspect of participant experience within the TCC. It is plausible that this reflects the flexibility that participants valued. Within the preliminary study of group Unified Protocol (Bullis et al., 2015), participants were reported as generally expressing that the diagnostic heterogeneity of the group was positive, with one participant saying they would value more targeted advice. This was a small study, and qualitative results are not provided in enough numerical detail to clearly ascertain how representative reported participant comments were. A flexible delivery style is not explicitly described as an aspect of the Unified Protocol or transdiagnostic CBT anxiety groups, and without more detailed qualitative data, it is difficult to ascertain whether participants felt groups were sufficiently adapted to their individual needs.

The TCC was designed so it could be offered to large groups, but the degree of flexibility would be constrained in this format. It could be that without this flexibility, participants might feel that the TCC was not sufficiently tailored to them, and this could affect acceptability. Further empirical work would be required to establish whether the individualized worksheets and experiential exercises of the TCC would offset this in a large group. However, it is also possible that flexibility could be offered in other ways. For example, three participants said that they would have liked more TCC sessions, and this corresponds to increasing evidence that psychological change does not occur within a predictable time frame and that psychological interventions can be offered that respond to differences in “change trajectory” (Carey & Spratt, 2009; Morris, Mansell, Emsley, et al., 2016). Although TCC sessions cover a range of topics and will be sufficient for many clients, for others, more (or less) therapy may be required. Similarly, although the aforedescribed studies of Unified Protocol and transdiagnostic group CBT offered more sessions than the TCC, a number of participants were reported to request additional treatment following the groups (Bullis et al., 2015; Norton & Hope, 2008). Due to the differences in study design, it is not appropriate to draw a detailed comparison, but it is possible that providing services that offer a range of psychological interventions in a flexible manner will better respond to the needs of individuals across psychological interventions. For example, one way in which such flexibility of access could be enhanced within the TCC is that clients could be given the option of joining sessions of a future course if they felt they needed a recap. It is already acknowledged that each individual will not attend every session; clients are provided with advance details of session content and are advised that they can choose not to attend every session.

Some of the aspects that participants valued are unlikely to be specific to the TCC; for example, “supporting each other” and normalization of experience are commonly described within group sessions (Malpass, Mansell, Emsley, et al., 2012). For example, within the preliminary study of group Unified Protocol (Bullis et al., 2015), some participants expressed finding it helpful hearing the experiences of others. Further, within the two case studies reported of participants who accessed a transdiagnostic CBT anxiety group, both these clients expressed that the group process was helpful (e.g., seeing other people coping with similar problems). However, the fact that that self‐disclosure was not required within the TCC, and could be paced, was also greatly valued and it is unclear how much this is a feature of other transdiagnostic groups.

### 4.2 Participants' experience of psychological change

If the theoretical premise of the TCC is well‐grounded, then this should be reflected in participant experience. Furthermore, due to the limited qualitative studies of transdiagnostic interventions, it is useful to establish if targeting transdiagnostic processes has face validity with clients.

Key themes of the TCC include acting in a flexible manner in order to meet personally important goals, and awareness; these featured strongly within participants' accounts. For example, participants described taking flexible steps to achieve long‐term (higher level) goals by aiming for short‐term achievable goals in pursuit of long‐term goals (Morris, Mansell, & McEvoy, 2016). Awareness techniques facilitated this process by enabling participants to focus on higher level goals and to manage thinking/behaviour that was preventing them from pursuing such goals.

Notably, however, references to conflict were generally absent from participants' accounts. Previous qualitative studies suggest that describing problems in terms of conflict is unusual, whereas describing problems in terms of loss of control is common (reviewed in Alsawy et al., 2014). Further, through identifying and pursuing important higher level goals despite the consequences of conflict (e.g., distress and intrusive imagery), conflict may be reduced and control regained (Carey, 2011). It is likely that conscious awareness of “conflict” is not always necessary for change (Alsawy et al., 2014). Further, a number of qualitative studies examining change processes have indicated that participants could identify what changed for them (such as thinking processes and perspectives) and specific techniques that were helpful but were not able to identify how their distress had been transformed (Marken & Carey, 2014). Despite limited reference to conflict within participants' accounts, generally, findings indicated that a strong theoretical basis can provide an accessible and consistent therapeutic approach; themes of control and awareness were strongly represented.

A focus on “control” is central to the TCC, and although the majority of participants found this helpful, a minority expressed difficulties. Participants who found it difficult to “let go” of “dwelling on” concerns that they could not control had all experienced significant life events or chronic ongoing problems during the TCC. Three out of the four scored in the severe range for both depression and anxiety. However, there were other participants who experienced significant events or chronic problems and did not express difficulties letting go of concerns they could not control. Therefore, it could be that the problems, or low mood/anxiety, experienced by those who struggled were too significant for them to either “let go” of dwelling upon their problems or take steps to control. In some instances, it seemed that participants were striving for an unachievable level of control and feeling responsible for things that they could not influence. To further support participants, it could be made even more explicit that they can break down goals that seem difficult to achieve, so they can focus on more achievable subgoals. Further, a discussion of control prior to the course could help to establish whether clients were aiming for an achievable level of control, and to explore this if not.

Aspects of the “Control” theme overlapped with themes found in qualitative studies of mindfulness‐based groups (Malpass, Mansell, Emsley, et al., 2012). Themes in such studies included letting go of what is outside your control and responding instead of reacting. Such themes or descriptions were not apparent in qualitative and case study data regarding group Unified Protocol and transdiagnostic anxiety CBT (Bullis et al., 2015; Norton & Hope, 2008). Increasing awareness is a significant component of the TCC, and brief mindfulness exercises are used. Further, PCT specifies mechanisms that are common across disorders; TCC targets these mechanisms overtly, but some other therapies (e.g., mindfulness‐based ones) are likely to also address these processes to varying degrees (Morris, Mansell, & McEvoy, 2016). Other themes were distinct, for example, the subthemes of “flexibility and an at ease environment” and “pursuing what really matters.”

In line with previous studies (Alsawy et al., 2014), participants reported both gradual and sudden changes. Only seven participants commented on this, five described “gradual change,” and three “sudden change” (one participant described both). Gradual change was more common and may reflect a PCT account that “changes at the higher level of the hierarchy are accompanied or preceded by changes in lower‐order systems that may not lead to significant change on their own” (Higginson & Mansell, 2008, p. 325). An example of this was provided in Section 1. However, PCT‐based accounts of significant change would generally predict a greater prevalence of reports of sudden changes, or “insight moments,” than described in the current study (Gianakis & Carey, 2011). This may be explained by “pace of change” not being a primary focus of the interviews; therefore, participants were not consistently asked in detail about this.

### 4.3 Limitations and implications

The primary limitation of this study was that those who attended low numbers of TCC sessions were under‐represented, and this could mean that those who did not find TCC useful could be under‐represented. However, there was some range in the number of sessions that participants attended (three to six) and considerable range in whether their symptoms had improved. Another limitation is that, although two researchers conducted the interviews, only the first author conducted the coding. Coding was examined by one of the project team (Dr Edge), but formal reliability checks were not conducted. A further possible limit is the sample size. However, the sample was relatively homogenous and saturation of codes occurred early (after the first eight interviews were coded).

Findings suggest some refinements to the TCC: (a) making it even more explicit that participants can break down goals that seem difficult to achieve, so they can focus on more achievable subgoals; (b) framing the exposure exercise to make it very clear that participants do not have to do this; and (c) supporting clients to choose manageable experiences during exposure by providing them with additional opportunities to reflect on the most suitable experience to choose.

The findings also have implications for other group‐based interventions of this kind. In particular, the format of the group should be clearly explained in advance owing to concerns that self‐disclosure will be required; any components that can be adapted to the individual tend to be valued, and more sessions could be provided where requested. Findings indicate the value of providing flexible and individualized treatment even within a group format. This has been identified in other settings (e.g., family therapy and one‐to‐one; Carey, 2005; Seikkula, 2002) but can provide a particular challenge in group settings while also offering a manualized and coherent treatment. The challenge here is to offer an intervention with a specific form, that is, replicable and evidence based, but with the potential to be adapted to both specific groups and individuals within these groups. Further qualitative research is required to examine whether the methods used to achieve this in the TCC (e.g., weekly feedback used to make adaptations, core content alongside optional or adaptable content, flexible ethos of facilitators, and curious questioning of individuals) would be similarly effective in creating an atmosphere of flexibility other groups (see Morris et al., in press, for a detailed description of such methods and how to deliver TCC).

## 5 CONCLUSIONS

Results indicated that the TCC was acceptable and provide an initial indication that transdiagnostic groups are acceptable. The strong theoretical basis seemed to provide an accessible transdiagnostic format.

## CONFLICT OF INTEREST

The authors confirm that there are no known conflicts of interest associated with this research project.

## APPENDIX A SEMISTRUCTURED INTERVIEW SCHEDULE

### A.1

This meeting today is to discuss you how found the Take Control Course. To help us improve the therapy I'll be asking you questions to find out if there were parts you found useful and to find out if there was anything less useful.

Before we begin have you got any questions?

**Attendance/Client experience of course**

1. Just thinking back to when you did the Take Control Course, how many sessions did you come to?
2. What did you hope to get out of the Take Control Course?
3. Do you remember any particular topics that were covered?

*Prompt: if cannot remember the sessions show them the structure of sessions, or provide a brief prompts as to content covered if appropriate*.

4. How did you find the Take Control Course sessions?

**Helpful and unhelpful aspects of sessions/course**

5. Was there anything about the sessions you found helpful?

*Potential follow‐up questions: what aspects did you find helpful? What made it “helpful”?*

6. It sounds like you found xxxx helpful what was it like using xxx?

*Potential follow‐up question: How much have you used xxx? (Daily, weekly, rarely)? Have you used xxx to deal with problems that have arisen for you in the past 6 months?*

7. Was there anything that wasn't helpful?

*(If participant seems reluctant to identify things are unhelpful, consider rewording, e.g. any bits that didn't appeal so much to you, that didn't make so much sense. Also consider reassuring them that all feedback will help us develop the course)*

*Potential follow‐up questions: What aspects did you not find helpful? ‘What made “unhelpful”*

**Changes in thinking**

8. Has anything changed in the way you deal with problems in your day to day life?

*Potential follow‐up questions: in what way do you deal with things now? How has the way you deal with problems stayed the same? Has anything changed in how you are with people?*

**Session Length/Research study**

9. What did you think about the amount of time the sessions lasted for?

*Potential follow‐up questions: How long would you have liked? Did you feel you had enough sessions?*

10. Do you think it would have made a difference if you went to the one‐to‐one sessions?

*Potential follow‐up questions: How different do you think it might have been if you had gone to the course/one‐to‐one session?*

**Research Study**

I've been asking about the Take Control Course and would also like to know more about your experience being part of a research project. Do you remember being part of a research study?

11. How was it taking part in a research project?

*Follow‐up questions: Do you remember how you ended up in the group? What do you think of the way that happened? Could it have been done differently?*

## References

[cpp2303-bib-0001] Aillon, J.‐L. , Ndetei, D. M. , Khasakhala, L. , Ngari, W. N. , Achola, H. O. , Akinyi, S. , & Ribero, S. (2014). Prevalence, types and comorbidity of mental disorders in a Kenyan primary health centre. Social Psychiatry and Psychiatric Epidemiology, 49(8), 1257–1268. 10.1007/s00127-013-0755-2 23959589

[cpp2303-bib-0002] Alsawy, S. , Mansell, W. , Carey, T. A. , McEvoy, P. , & Tai, S. J. (2014). Science and practice of transdiagnostic CBT: A perceptual control theory (PCT) approach. International Journal of Cognitive Therapy, 7(4), 334–359. 10.1521/ijct.2014.7.4.334

[cpp2303-bib-0003] Andersson, G. , & Titov, N. (2014). Advantages and limitations of Internet‐based interventions for common mental disorders. World Psychiatry, 13(1), 4–11. 10.1002/wps.20083 24497236 PMC3918007

[cpp2303-bib-0004] Angen, M. J. (2000). Evaluating interpretive inquiry: Reviewing the validity debate and opening the dialogue. Qualitative Health Research, 10(3), 378–395. 10.1177/104973230001000308 10947483

[cpp2303-bib-0005] Ayala, G. X. , & Elder, J. P. (2011). Qualitative methods to ensure acceptability of behavioral and social interventions to the target population. Journal of Public Health Dentistry, 71(s1), S69–S79. 10.1111/j.1752-7325.2011.00241.x 21656958 PMC3758883

[cpp2303-bib-0006] Barlow, D. H. , Allen, L. B. , & Choate, M. L. (2004). Toward a unified treatment for emotional disorders. Behavior Therapy, 35(2), 205–230. 10.1016/S0005-7894(04)80036-4 27993336

[cpp2303-bib-0007] Bennett‐Levy, J. , Richards, D. , & Farrand, P. (2010). Low intensity CBT interventions: A revolution in mental health care In Bennett‐Levy J., Richards D., Farrand P., Christensen H., Griffiths K., Kavanagh D., et al. (Eds.), Oxford guide to low intensity CBT interventions (pp. 3–18). Oxford: Oxford University Press.

[cpp2303-bib-0008] Bower, P. , & Gilbody, S. (2005). Stepped care in psychological therapies: Access, effectiveness and efficiency. Narrative literature review. The British Journal of Psychiatry, 186(1), 11–17. 10.1192/bjp.186.1.11 15630118

[cpp2303-bib-0009] Braun, V. , & Clarke, V. (2006). Using thematic analysis in psychology. Qualitative Research in Psychology, 3(2), 77–101. 10.1191/1478088706qp063oa

[cpp2303-bib-0010] Bullis, J. R. , Sauer‐Zavala, S. , Bentley, K. H. , Thompson‐Hollands, J. , Carl, J. R. , & Barlow, D. H. (2015). The unified protocol for transdiagnostic treatment of emotional disorders: Preliminary exploration of effectiveness for group delivery. Behavior Modification, 39(2), 295–321. 10.1177/0145445514553094 25316034

[cpp2303-bib-0011] Burns, P. , Kellett, S. , & Donohoe, G. (2016). “Stress Control” as a large group psychoeducational intervention at Step 2 of IAPT services: Acceptability of the approach and moderators of effectiveness. Behavioural and Cognitive Psychotherapy, 44(04), 431–443. 10.1017/S1352465815000491 26365006

[cpp2303-bib-0012] Carey, T. A. (2005). Can patients specify treatment parameters? A preliminary investigation. Clinical Psychology & Psychotherapy, 12(4), 326–335. 10.1002/cpp.455

[cpp2303-bib-0013] Carey, T. A. (2011). Exposure and reorganization: The what and how of effective psychotherapy. Clinical Psychology Review, 31(2), 236–248. 10.1016/j.cpr.2010.04.004 20447745

[cpp2303-bib-0014] Carey, T. A. , & Spratt, M. B. (2009). When is enough enough? Structuring the organisation of treatment to maximise patient choice and control. The Cognitive Behaviour Therapist, 2, 211–226. 10.1017/S1754470X09000208

[cpp2303-bib-0015] Carlson, J. A. (2010). Avoiding traps in member checking. The Qualitative Report, 15(5), 1102.

[cpp2303-bib-0016] Carryer, J. R. , & Greenberg, L. S. (2010). Optimal levels of emotional arousal in experiential therapy of depression. Journal of Consulting and Clinical Psychology, 78(2), 190 10.1037/a0018401 20350030

[cpp2303-bib-0017] Chambers, D. A. , Haim, A. , Mullican, C. A. , & Stirratt, M. (2013). Health information technology and mental health services research: A path forward. General Hospital Psychiatry, 4(35), 329–331. 10.1016/j.genhosppsych.2013.03.006 23582190

[cpp2303-bib-0018] Chiesa, A. , Calati, R. , & Serretti, A. (2011). Does mindfulness training improve cognitive abilities? A systematic review of neuropsychological findings. Clinical Psychology Review, 31(3), 449–464. 10.1016/j.cpr.2010.11.00 21183265

[cpp2303-bib-0019] Cho, J. , & Trent, A. (2006). Validity in qualitative research revisited. Qualitative Research, 6(3), 319–340. 10.1177/1468794106065006

[cpp2303-bib-0020] Creswell, J. W. (2009). Research design: Qualitative, quantitative, and mixed methods approaches. London, UK: SAGE Publications, Incorporated.

[cpp2303-bib-0105] Department of Communities and Local Government (2011). The English indices of deprivation 2010. Retrieved from http://www.communities.gov.uk/publications/corporate/statistics/ indices2010

[cpp2303-bib-0021] Farb, N. A. , Anderson, A. K. , Irving, J. A. , & Segal, Z. (2014). Mindfulness interventions and emotion regulation In Gross J. J. (Ed.), Handbook of emotion regulation (pp. 548–567). New York: Guilford Press.

[cpp2303-bib-0022] Finucane, A. , & Mercer, S. W. (2006). An exploratory mixed methods study of the acceptability and effectiveness of mindfulness‐based cognitive therapy for patients with active depression and anxiety in primary care. BMC Psychiatry, 6(1), 14 10.1186/1471-244X-6-14 16603060 PMC1456957

[cpp2303-bib-0023] Gianakis, M. , & Carey, T. A. (2011). An interview study investigating experiences of psychological change without psychotherapy. Psychology and Psychotherapy: Theory, Research and Practice, 84(4), 442–457. 10.1111/j.2044-8341.2010.02002.x 22903885

[cpp2303-bib-0024] Gu, J. , Strauss, C. , Bond, R. , & Cavanagh, K. (2015). How do mindfulness‐based cognitive therapy and mindfulness‐based stress reduction improve mental health and wellbeing? A systematic review and meta‐analysis of mediation studies. Clinical Psychology Review, 37, 1–12. 10.1016/j.cpr.2015.01.006 25689576

[cpp2303-bib-0025] Guest, G. , Bunce, A. , & Johnson, L. (2006). How many interviews are enough? An experiment with data saturation and variability. Field Methods, 18(1), 59–82. 10.1177/1525822X05279903

[cpp2303-bib-0026] Higginson, S. , & Mansell, W. (2008). What is the mechanism of psychological change? A qualitative analysis of six individuals who experienced personal change and recovery. Psychology and Psychotherapy‐Theory Research and Practice, 81, 309–328. 10.1348/147608308x320125 18588749

[cpp2303-bib-0027] Khan, N. , Bower, P. , & Rogers, A. (2007). Guided self‐help in primary care mental health: Meta‐synthesis of qualitative studies of patient experience. The British Journal of Psychiatry, 191(3), 206–211. 10.1192/bjp.bp.106.032011 17766759

[cpp2303-bib-0028] Knowles, S. E. , Toms, G. , Sanders, C. , Bee, P. , Lovell, K. , Rennick‐Egglestone, S. , … Kessler, D. (2014). Qualitative meta‐synthesis of user experience of computerised therapy for depression and anxiety. PLoS one, 9(1). e84323. doi: 10.1371/journal.pone.0084323 PMC3894944 24465404

[cpp2303-bib-0029] Lewis, C. , Pearce, J. , & Bisson, J. I. (2012). Efficacy, cost‐effectiveness and acceptability of self‐help interventions for anxiety disorders: Systematic review. The British Journal of Psychiatry, 200(1), 15–21. 10.1192/bjp.bp.110.084756 22215865

[cpp2303-bib-0030] Lippelt, D. P. , Hommel, B. , & Colzato, L. S. (2014). Focused attention, open monitoring and loving kindness meditation: Effects on attention, conflict monitoring, and creativity—A review. Frontiers in Psychology, 5 10.3389/fpsyg.2014.01083 PMC4171985 25295025

[cpp2303-bib-0031] Macdonald, W. , Mead, N. , Bower, P. , Richards, D. , & Lovell, K. (2007). A qualitative study of patients' perceptions of a ‘minimal’ psychological therapy. International Journal of Social Psychiatry, 53(1), 23–35. 10.1177/0020764006066841 17333949

[cpp2303-bib-0032] Malpass, A. , Carel, H. , Ridd, M. , Shaw, A. , Kessler, D. , Sharp, D. , … Wallond, J. (2012). Transforming the perceptual situation: A meta‐ethnography of qualitative work reporting patients' experiences of mindfulness‐based approaches. Mindfulness, 3(1), 60–75. 10.1007/s12671-011-0081-2

[cpp2303-bib-0033] Mansell, W. , Harvey, A. , Watkins, E. , & Shafran, R. (2009). Conceptual foundations of the transdiagnostic approach to CBT. Journal of Cognitive Psychotherapy, 23(1), 6–19. 10.1891/0889-8391.23.1.6

[cpp2303-bib-0034] Marken, R. S. , & Carey, T. A. (2014). Understanding the change process involved in solving psychological problems: A model‐based approach to understanding how psychotherapy works. Clinical Psychology & Psychotherapy, 22, 580–590. 10.1002/cpp.1919 25219953

[cpp2303-bib-0035] Morris, L. , & Mansell, W. (in press). A systematic review of the relationship between rigidity/flexibility and transdiagnostic cognitive and behavioral processes that maintain psychopathology. Journal of Experimental Psychopathology.

[cpp2303-bib-0036] Morris, L. , Mansell, W. , Emsley, R. , Bates, R. , Comiskey, J. , Pistorius, E. , & McEvoy, P. (2016). Prospective cohort study of a transdiagnostic group intervention for common mental health problems: The Take Control Course. Psychology and Psychotherapy‐ Theory Research and Practice, 89, 163–180. 10.1111/papt.12070 26200798

[cpp2303-bib-0037] Morris, L. , Mansell, W. , & McEvoy, P. (2016). The Take Control Course: Conceptual rationale for the development of a transdiagnostic group for common mental health problems. Frontiers in Psychology, 7(99). 10.3389/fpsyg.2016.00099 PMC4748307 26903907

[cpp2303-bib-0038] Morris, L. , McEvoy, P. , Wallwork, W. , Bates, R. , Comiskey, J. , & Mansell, W. (in press). Transdiagnostic group therapy training and implementation: The Take Control Course. San Diego, CA: Elsevier.

[cpp2303-bib-0039] Morse, J. M. , Barrett, M. , Mayan, M. , Olson, K. , & Spiers, J. (2002). Verification strategies for establishing reliability and validity in qualitative research. International Journal of Qualitative Methods, 1(2), 13–22. 10.1177/160940690200100202

[cpp2303-bib-0040] Newby, J. M. , McKinnon, A. , Kuyken, W. , Gilbody, S. , & Dalgleish, T. (2015). Systematic review and meta‐analysis of transdiagnostic psychological treatments for anxiety and depressive disorders in adulthood. Clinical Psychology Review, 40, 91–110. 10.1016/j.cpr.2015.06.002 26094079

[cpp2303-bib-0041] Norton, P. J. , & Hope, D. A. (2008). The “anxiety treatment protocol”: A group case study demonstration of a transdiagnostic group cognitive–behavioral therapy for anxiety disorders. Clinical Case Studies, 7(6), 538–554. 10.1177/1534650108321307

[cpp2303-bib-0106] Office for National Statistics (2011). Neighbourhood statistics: Resident population estimates byethnic group (2009 estimates). Retrieved from http://www.neighbourhood.statistics.co.uk

[cpp2303-bib-0100] Onwuegbuzie, A. J. , & Leech, N. L. (2007). Sampling Designs in Qualitative Research: Making the Sampling Process More Public. The Qualitative Report, 12(2), 238–254. Retrieved from http://nsuworks.nova.edu/tqr/vol12/iss2/7

[cpp2303-bib-0042] Powers, W. T. (1973). Behavior: The control of perception. Chicago, IL: Aldine.

[cpp2303-bib-0043] QSR International's NVivo 10 Software (2014). Qualitative data analysis software. QSR International Pty Ltd. https://www.qsrinternational.com/nvivo/support-overview/faqs/how-do-i-cite-nvivofor-mac,-nvivo-11-for-windows

[cpp2303-bib-0110] Richards, D. A. , & Suckling, R. (2009). Improving access to psychological therapies: Phase IV prospective cohort study. British Journal of Clinical Psychology, 48, 377–396. https://doi:10.1348/014466509X405178 19208291 10.1348/014466509X405178

[cpp2303-bib-0044] Richards, D. A. (2010). Access and organization: Putting low intensity interventions to work in clinical services In Bennett‐Levy J., Richards D. A., Farrand P., Christensen H., Griffiths K., Kavanagh D., et al. (Eds.), Oxford guide to low intensity CBT interventions. New York: Oxford University Press.

[cpp2303-bib-0045] Sanders, C. , Rogers, A. , Bowen, R. , Bower, P. , Hirani, S. , Cartwright, M. , … Hendy, J. (2012). Exploring barriers to participation and adoption of telehealth and telecare within the Whole System Demonstrator trial: A qualitative study. BMC Health Services Research, 12(1), 220 10.1186/1472-6963-12-220 22834978 PMC3413558

[cpp2303-bib-0046] Seikkula, J. (2002). Open dialogues with good and poor outcomes for psychotic crises: Examples from families with violence. Journal of Marital and Family Therapy, 28(3), 263–274. 10.1111/j.1752-0606.2002.tb01183.x 12197148

[cpp2303-bib-0047] Shapero, B. G. , Greenberg, J. , Mischoulon, D. , Pedrelli, P. , Meade, K. , & Lazar, S. W. (2018). Mindfulness‐based cognitive therapy improves cognitive functioning and flexibility among individuals with elevated depressive symptoms. Mindfulness, 1–13. 10.1007/s12671-018-0889-0

[cpp2303-bib-0048] Shenton, A. K. (2004). Strategies for ensuring trustworthiness in qualitative research projects. Education for Information, 22(2), 63–75. 10.3233/EFI-2004-22201

[cpp2303-bib-0049] Teper, R. , Segal, Z. V. , & Inzlicht, M. (2013). Inside the mindful mind: How mindfulness enhances emotion regulation through improvements in executive control. Current Directions in Psychological Science, 22(6), 449–454. 10.1177/0963721413495869

[cpp2303-bib-0050] Tuckett, A. (2004). Qualitative research sampling: The very real complexities. Nurse Researcher, 12(1), 47–61. 10.7748/nr2004.07.12.1.47.c5930 15493214

[cpp2303-bib-0051] White, J. (2010). The STEPS model: A high volume, multi‐level, multi‐purpose approach to address common mental health problems In Bennett‐Levy J., Richards D., Farrand P., Christensen H., Griffiths K., Kavanagh D., et al. (Eds.), Oxford guide to low intensity CBT interventions. New York: Oxford University Press.

[cpp2303-bib-0052] White, J. , Keenan, M. , & Brooks, N. (1992). Stress control: A controlled comparative investigation of large group therapy for generalized anxiety disorder. Behavioural Psychotherapy, 20(2), 97–113. 10.1017/S014134730001689X

[cpp2303-bib-0053] Wilamowska, Z. A. , Thompson‐Hollands, J. , Fairholme, C. P. , Ellard, K. K. , Farchione, T. J. , & Barlow, D. H. (2010). Conceptual background, development, and preliminary data from the unified protocol for transdiagnostic treatment of emotional disorders. Depression and Anxiety, 27(10), 882–890. 10.1002/da.20735 20886609

